# Computer-Aided Drug Design Using Sesquiterpene Lactones as Sources of New Structures with Potential Activity against Infectious Neglected Diseases

**DOI:** 10.3390/molecules22010079

**Published:** 2017-01-03

**Authors:** Chonny Herrera Acevedo, Luciana Scotti, Mateus Feitosa Alves, Margareth De Fátima Formiga Melo Diniz, Marcus Tullius Scotti

**Affiliations:** Post-Graduate Program in Natural and Synthetic Bioactive Products, Federal University of Paraíba, 58051-900 João Pessoa, PB, Brazil; caherrera@ltf.ufpb.br (C.H.A.); luciana.scotti@gmail.com (L.S.); mateusfalves@gmail.com (M.F.A.); margareth@ltf.ufpb.br (M.D.F.F.M.D.)

**Keywords:** computer-aided drug design, neglected tropical diseases, sesquiterpene lactones, secondary metabolites, leishmaniasis, Chagas disease, sleeping sickness, schistosomiasis, QSAR studies

## Abstract

This review presents an survey to the biological importance of sesquiterpene lactones (SLs) in the fight against four infectious neglected tropical diseases (NTDs)—leishmaniasis, schistosomiasis, Chagas disease, and sleeping sickness—as alternatives to the current chemotherapies that display several problems such as low effectiveness, resistance, and high toxicity. Several studies have demonstrated the great potential of some SLs as therapeutic agents for these NTDs and the relationship between the protozoal activities with their chemical structure. Recently, Computer-Aided Drug Design (CADD) studies have helped increase the knowledge of SLs regarding their mechanisms, the discovery of new lead molecules, the identification of pharmacophore groups and increase the biological activity by employing in silico tools such as molecular docking, virtual screening and Quantitative-Structure Activity Relationship (QSAR) studies.

## 1. Introduction

Neglected tropical diseases (NTDs) are diseases that are present around the world in tropical and subtropical regions. They affect about one billion people, who commonly live in poverty [1], from 150 countries, causing some 140,000 deaths per year [2]. These types of diseases are attributable to inadequate access to safe water, sanitation, and appropriate housing [3], which presents difficulties in their prevention, control, and elimination, and generate costs of billions of dollars a year in those countries [1].

According to the classification of the World Health Organization, 19 NTDs exist [4], of which eight—Buruli ulcer, trachoma, leprosy, rabies, endemic treponematoses, mycetoma, dengue, and chikungunya—are caused by viruses or bacteria, while 11 others—dracunculiasis, soil-transmitted helminthiases, echinococcosis, foodborne trematodiases, lymphatic filariasis, onchocerciasis, taeniasis, schistosomiasis, American trypanosomiasis (Chagas disease), human African trypanosomiasis (sleeping sickness), and leishmaniasis—are classified as parasitic diseases. The last three, which are generated by different kinetoplastic parasite species [5], have more impact worldwide due to the approximately two million new cases and more than 50,000 deaths annually, which combined with schistosomiasis are responsible for about 60% of all the deaths resulting from NTDs [2,6].

Antimonial compounds are the main treatment against leishmaniasis, which presents high toxicity and resistance in some endemic regions. Nifurtimox and benznidazole, the drugs employed against *Trypanosoma cruzi*, present many side effects in patients. The low effectivity in the chronic phase of the disease, the high risk of death from melarsoprol, an arsenic derivative for treating the chronic phase in human African trypanosomiasis, and the increase in cases of resistance of schistosoma to praziquantel treatment are some examples of chemotherapy actually used for NTDs that present several problems and prevent the control and elimination of these diseases.

Because of this, it is necessary to discover new low cost drugs that are highly effective with low toxicity. Humans have thus turned to plants as a source of compounds for the treatment of many diseases, and in addition to being an option to find solutions for many health problems of most of the world’s population, is an important industry, as plants generate over $25 billion dollars per year in value based only on the relevant pharmaceuticals derived from plants [7].

One of the most studied plant families in the natural products research field is the Asteraceae (Compositae). It is the largest family of flowering plants, formed by more than 23,000 species that are present in every continent, principally in the New World [8]. Some molecules found in this family have been successfully used in humans against parasite diseases, such artemisinin, an antimalarial sesquiterpene lactone which discovery led to the 2015 Nobel Prize for Medicine and Physiology [9,10]. This drug, which has the most rapid action against the malaria caused by *Plasmodium falciparum*, opened the panorama with respect to research into new therapeutics for leishmaniasis, trypanosomiasis, and schistosomiasis from this type of secondary metabolite, and in addition to the antimalarial activity, the drug has antitumor potential and shows prevention of neurodegeneration, antimigraine activity, analgesic, and sedative activities [11].

In recent years, cheminformatics tools have served in the taxonomic classification of the sesquiterpene lactones [12,13,14], cytotoxicity assays [15,16,17], as well as the continuous search for new drugs or lead compounds through the development of new methodologies in Computer-Aided Drug Design (CADD). These in silico studies have emerged as a good alternative in medicinal chemistry for helping to establish the experimental design, find new drugs, and compare the molecular structure and activity presented through one of the most important tools in this area, quantitative structure–activity relationship (QSAR) studies, generating successful results with low cost in a short time.

In brief, this review summarizes the principal available information with respect to the sesquiterpene lactones in the search for effective and safe drugs against four infectious NTDs (leishmaniasis, human African trypanosomiasis, Chagas disease, and schistosomiasis), highlighting the corresponding QSAR studies.

## 2. The Current Chemotherapy against Infectious NTDs

### 2.1. Leishmaniasis

Leishmaniases are anthroponotic and zoonotic diseases caused by approximately 20 protozoan parasite species of the genus *Leishmania*, which are transmitted to humans by more than 30 different species of phlebotomine sandflies [18,19]. In accordance with the clinical manifestations, the three main forms of leishmaniasis are cutaneous, mucocutaneous, and visceral [19,20]. Visceral leishmaniasis or kala-azar is the most severe form of the disease, being fatal in the absence of treatment [21], and some studies report that even 5%–10% of patients under medication die [22].

**Figure 1:**
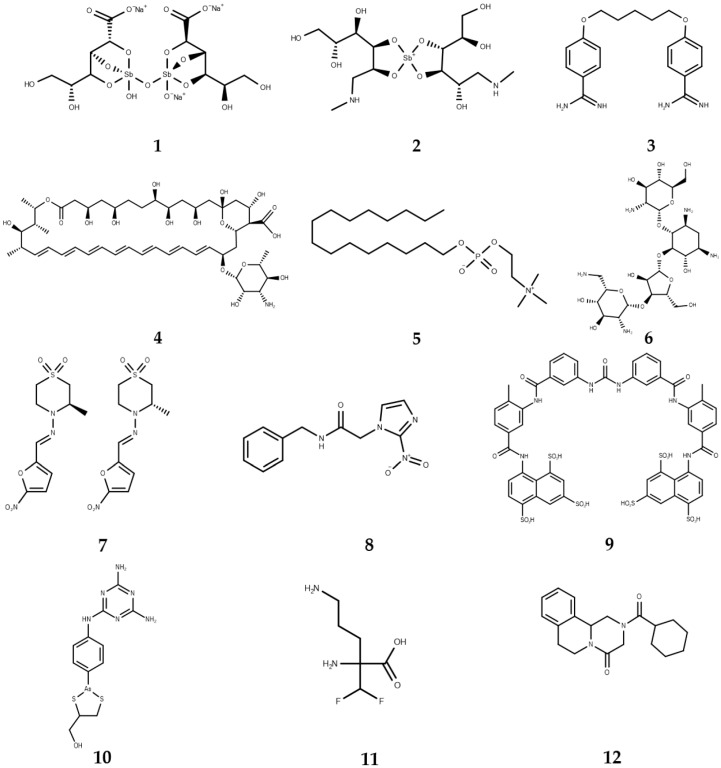
Chemotherapy used against four neglected tropical diseases.

For over five decades, the main treatment has consisted of pentavalent antimonial compounds such as sodium stibogluconate (**1**, Figure 1) and meglumine antimoniate (**2**, Figure 1), the efficacy of which is between 80% and 95% [23,24,25]. They are low cost antileishmanial drugs, easily available in endemic regions [26], however, resistance is observed in some regions [27,28], and toxicity [29] and the long duration of treatment (up to 28 days of parenteral administration) [30] have emerged as the principal problems for the use of those drugs, which has prompted the search for new compounds. Another drug, pentamidine (**3**, Figure 1), initially used in India for visceral leishmaniasis, was discontinued due to the many adverse reactions related with its use, however, it was recently re-introduced in Ethiopia for the treatment of HIV–cutaneous leishmaniasis co-infections [26]. Amphotericin B (**4**, Figure 1) presents the highest effectivity, but the treatment requires prolonged hospitalization in addition to producing side effects such as fever, vomiting, abdominal pain, and nephrotoxicity. The liposomal form is less toxic, but both forms are very expensive [19,20]. Miltefosine (**5**, Figure 1) and paromomycin (**6**, Figure 1), which have also been used recently as antileishmanial drugs, present several problems, such as toxicity (mainly renal), increased resistance, and teratogenic and abortifacient effects [31,32].

### 2.2. Trypanosomiasis

Trypanosomiasis is the name of the disease caused by a protozoan parasite of the genus *Trypanosoma*. Two types of trypanosomiasis can affect humans: Chagas disease (American trypanosomiases) and sleeping sickness (human African trypanosomiases).

Chagas disease is the highest impact parasitic disease in the Americas [33], affecting about 8 million persons, mainly in the poorest regions of Central and South America. It is attributed 10,600 deaths per year [2], usually as a result of heart failure or thromboembolic episodes. The infection is caused by the flagellated protozoan *Trypanosoma cruzi*, which is transmitted to humans by the *Triatoma* bug vector.

There is a limitation in the treatments currently used against Chagas disease as observed in leishmaniasis because several studies have shown inefficiency, resistance [34], toxic side effects [35], and reactivation of infections that mainly appear due to the increase of *T. cruzi*–HIV co-infections [36]. The chemotherapy of Chagas’ disease currently uses two drugs: nifurtimox (**7**, Figure 1) and benznidazole (**8**, Figure 1) [37]. The effectivity is close to 100%, but not in all cases. Various difficulties are presented with these drugs such as low rates of reduction of parasitemia in the chronic phase and mainly in the late phase of the disease, resistance of some strains of *T. cruzi*, and many side effects such as nausea, pain, and vomiting, in addition to an prolonged treatment time that in some cases can be up to two months. These difficulties have led to the continuous search for alternatives for the elimination of these diseases [37,38].

Sleeping sickness is transmitted by the bite of insects of the *Glossina* genus, also known as tsetse fly, which acts as a vector for different species of *Trypanosoma brucei* [39]. In 2014, 3796 cases were reported [40] and is an NTD mainly present in sub-Saharan Africa.

The treatment of sleeping sickness is divided in accordance with the disease phases, and only a few drugs with many side effects and limited efficacy are used. For the first phase, where the parasites are restricted to the hemolymphatic system, the above-mentioned pentamidine (**3**) together with suramin (**9**, Figure 1) are the drugs used against *T. brucei gambiense* and *T. brucei rhodiense*, respectively [41,42]. Both are relatively effective against the parasite, but they have side effects such as allergy, fatigue, neuropathy, renal problems, and nausea [42].

The treatment in the second phase presents more difficulties due to the fact *T. brucei* is present in the cerebrospinal fluid. For this reason, the drugs for the treatment in this stage must have the ability to cross the blood–brain barrier [41]. Melarsoprol (**10**, Figure 1) is an arsenic-derived compound that was for a long time the only compound available to treat patients at this stage of the disease. The drug has many adverse effects such as high risk of death, including liver toxicity, severe enterocolitis, diffuse peripheral neuropathy, and encephalopathic syndromes that occur in 2%–10% of patients, leading to death in half of these cases [41,43,44]. Eflornithine (**11**, Figure 1) is safer than melarsoprol and has emerged as an alternative because studies with patients in the second stage of human African trypanosomiasis presented better results with respect to the cutaneous and neurological adverse effects and death risk factor, but these positive results are only observed against *T. brucei gambiense* [44,45].

### 2.3. Schistosomiasis

Schistosomiasis is an NTD caused by trematode flukes of the genus *Schistosoma* spp., which affects more than 200 million people, most commonly in the sub-Saharan region in Africa [46]. The chronic infection causes anemia, neurological complications, intestinal fibrosis veins, hepatosplenomegaly, stunted growth, and other negative conditions, and this infection leads to approximately 5500 deaths each year [2,47]. It is anticipated for the morbidity caused by schistosomiasis will be controlled and eliminated as a public health problem in 2020 and 2025, respectively. To reach this goal, efforts in the social aspects of treatment such as sanitation, provision of water and hygiene facilities, and effective treatments for the elimination of parasites are necessary [46,48].

Praziquantel (**12**, Figure 1) is the drug that has been used in chemotherapy since 1970 for treating schistosomiasis and is almost the only available drug against this disease [49,50]. In contrast to the state of antileishmanial and antitrypanosomal treatments, praziquantel, besides its high efficacy, is safe and does not have toxicity problems [49], however, in the last two decades, some studies have shown schistosomiasis cases caused by *S. haematobium* infections in which repeated standard treatment fails to clear the infection, while *S. mansoni* presents a reduced susceptibility in field isolates and may develop resistance to schistosomicidal drugs over the course of relatively few passages [50,51,52].

**Figure 2:**
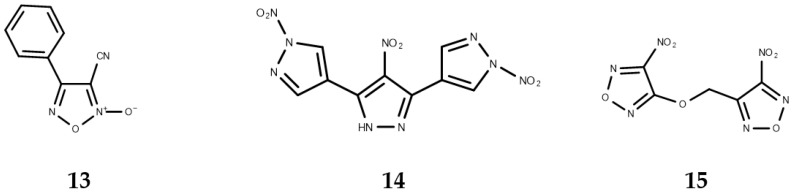
Oxadiazoles with antischistosomal activity.

**Figure 3:**
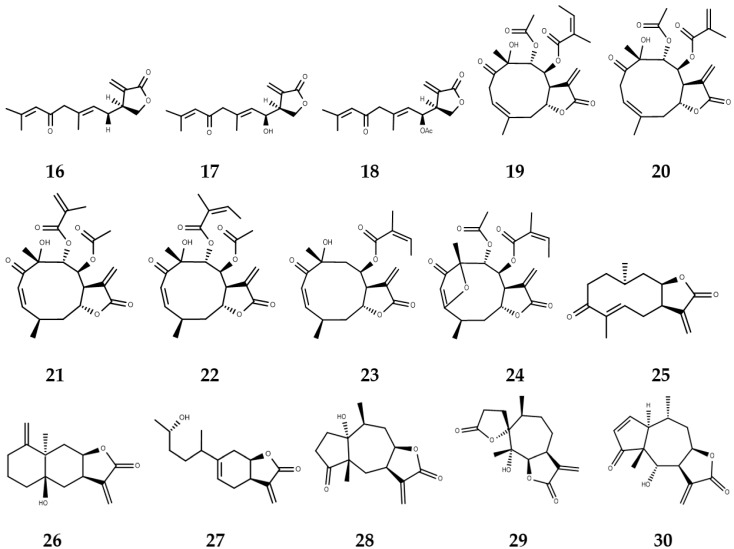

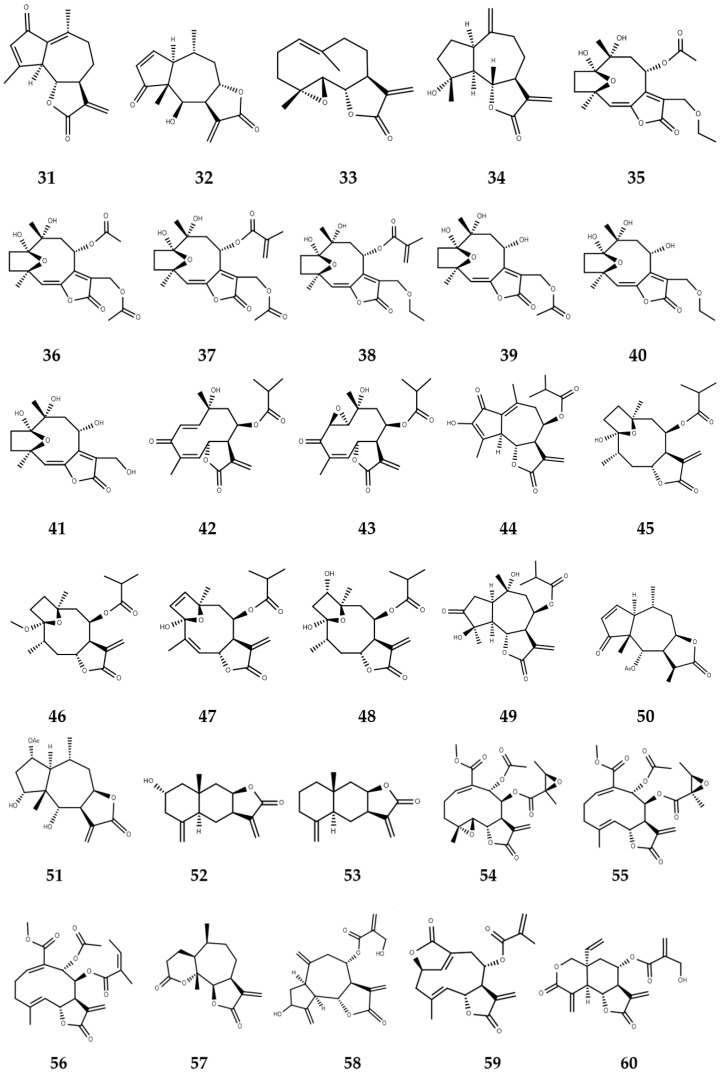

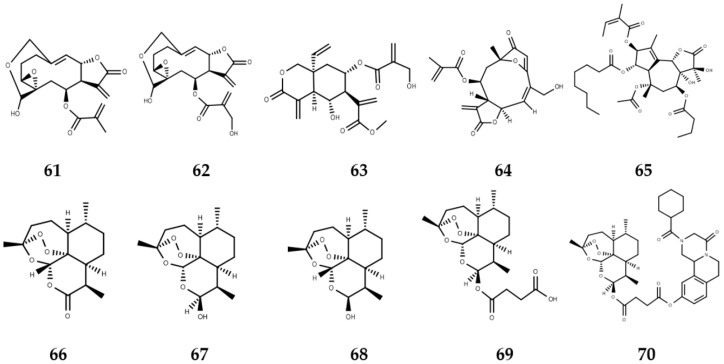
Sesquiterpene lactones with biological activity against NTDs.

Oxadiazoles have been studied in the search for alternative drugs against praziquantel-resistant parasites. Incubation of *S. mansoni* parasites with oxadiazole 2-oxides leads to parasite death by the inhibition of thioredoxin glutathione reductase (TGR), which is a crucial enzyme whose role is in the redox homeostasis of the schistosoma [53]. This antischistosomal activity was associated with the donation of nitric oxide [54]. This activity was evaluated in vivo, *S. mansoni*-infected mice were treated with 4-phenyl-1,2,5-oxadiazole-3-carbonitrile-2-oxide (**13**, Figure 2) and worm burden reductions of 99%, 88%, and 94% were observed from treatments against skin-, liver-, and adult-stage parasites, respectively [53]. In silico QSAR models using consensus analysis and virtual screening led to the proposal of two novel chemical scaffolds with antischistosomal activity: 4-nitro-3,5-bis(1-nitro-1*H*-pyrazol-4-yl)-1*H*-pyrazole (**14**, Figure 2) and 3-nitro-4-{[(4-nitro-1,2,5-oxadiazol-3-yl)oxy]meth-1,2,5-oxadiazole (**15**, Figure 2) [55].

## 3. Sesquiterpene Lactones with Activity against NTDs

### 3.1. Sesquiterpene Lactones with Leishmanicidal Activity

Several studies testing in vitro and in vivo activity of numerous SLs (Figure 3) have been performed in the search for new drugs against leishmaniasis. Axenic amastigotes of *L. donovani* were incubated with three anthecotulide-type linear SLs (anthecotulide (**16**), 4-hydroxyanthecotulide (**17**) and 4-acetoxyanthecotulide (**18**)) from *Anthemis auriculata* and a significant antileishmanial activity was observed; however, they are non-viable as future drugs due to the high in vitro cytotoxic activity values with mammalian L6 cells presented by the three molecules [56].

By evaluating antileishmanial activity against *L. donovani* looking for alternative drugs in leishmaniasis treatment, six germacranolide SLs **19**–**24** of *Calea zacatechichi* showed high IC_50_ values for this strain (IC_50_ as concentration that inhibits 50% of parasite growth), calealactone C (**20**) and calein D (**21**) (IC_50_ 1.9 and 2.2 μM, respectively) presented lower values than pentamidime (IC_50_ 2.9 μM), which is a drug used as positive control [57]. Inuloxins A, C, and D (**25**–**27**) (IC_50_ 6.9, 15.3, and 15.5 μM, respectively) also showed leishmanial activity for *L. donovani* promastigotes among 12 molecules tested, and the activity value of inuloxin A (**25**) was close to that of the positive control pentamidine (IC_50_ 4.8 μM), which is being proposed by the authors as a promising new antileishmanial lead [58].

Some SLs with antileishmanial activity for parasite forms of *L. mexicana* have been identified as promising therapies against leishmaniasis. By bioassay-guided fractionation, two SLs from *Ambrosia tenuifolia*, peruvin (**28**) and psilostachyin (**29**), besides the trypanocidal activity, showed low values of IC_50_ 1.5 μM and 0.4 μM, respectively, for the promastigote form [59]. Helenalin (**30**), together with dehydroleucodine (**31**) and mexicanin I (**32**), also have activity against *L. mexicana* promastigotes. For **30** and **32**, an IC_50_ value of 1.9 μM was obtained, while for **31**, it was 3.8 μM and these three drugs were more effective than ketoconazole (leishmanicidal). Low concentrations of these three SLs reduced the number of parasites per cell (Vero cells) and disturbed the growth capacity of the parasite [60]. The mechanism of these three was recently evaluated and show an increase in the generation of reactive oxygen species (ROS) by parasites and decrease the endogenous concentration of glutathione in the parasite, which has been associated with the induction of oxidative stress on promastigotes of *L. mexicana* [61].

Parthenolide (**33**) is a very common SL that is present in several species of the Asteraceae family. Tiuman et al. purified this compound from *Tanacetum parthenium*, which had previously been studied in promastigotes [62], and its leishmanial activity has been studied against axenic and intracellular amastigotes of *L. amazonensis* obtained after 72 h of incubation. IC_50_ values of 1.3 μM and 2.9 μM, respectively, were obtained, and these measurements were higher concentrations than those observed for the positive control amphotericin B, which presented an IC_50_ value of 0.22 μM. Using scanning electron microscopy (SEM), changes were observed in the parasite form, in vivo, and parthenolide concentrations of 4.0, 3.2, 2.4, and 1.6 μM reduced the proliferation of parasites into mouse macrophages and showed a survival index of 82.5%, 59.4%, 37.3%, and 6.1%, respectively [63].

In vitro and in vivo activity against *L. amazonensis* was tested from an SL-rich dichloromethane fraction of this species. IC_50_ values of 2.40 μg/mL and 1.76 μg/mL were obtained with promastigotes and axenic amastigotes, respectively. In vivo, it was observed that after four weeks of treatment with the dichloromethane fraction, a reduction in the growth and size of footpad lesions in BALB/c mice infected with promastigotes of *L. amazonensis* occurred. The main SLs present in the dichloromethane fraction are parthenolide (**33**) and 11,13-dehydrocompressanolide (**3**) and according to the differences between the plasma malondialdehyde (MDA) levels observed in the treated mice with respect to the control group, the lipid oxidation level increased; thus these SLs cause oxidative stress by producing free radicals, which include cellular apoptosis [64].

In other studies, the activity of seven hirsutinolide-type SLs **35**–**41** isolated from *Pseudelephantopus spiralis* was tested with promastigotes, axenic amastigotes, and intramacrophagic amastigotes (using J774A.1 cells) of *L. infantum*. The results show that the hirsutinolides are more active against the amastigote than against the promastigote form, and compound **37** presented the lowest IC_50_ values for these two parasite forms of 9.5 μM (promastigotes) and 2.0 μM (amastigotes). A low selectivity index, however, was obtained and all SLs tested were inactive in intramacrophagic amastigotes at the evaluated concentration [65]. Antileishmanial activities of eight SLs **42**–**49** were tested in amastigotes and promastigotes of *L. braziliensis* as well as the cytotoxic activity in macrophages. The results show that these SLs are promising leads due to the six of seven compounds evaluated presenting activity (only **44** was not effective) while being selective against the parasite; **46**–**48** were the most active against promastigotes and amastigotes, and **43** had the lowest LD_50_ (the 50% lethal dose: required dose for killing the 50% of parasites) value for promastigotes (6.0 μM) and the highest selective index (>22.7) [66].

### 3.2. Sesquiterpene Lactones with Trypanocidal Activity

Similarly, some SLs have shown trypanosomal activity and emerged as promising molecules for treatments against *T. cruzi* and/or *T. brucei*. For example, an in vitro study to evaluate the anti-*T. cruzi*, anti-*T. brucei rhodiense*, and cytotoxic activity (L-6 cells) of six SLs (helenalin, mexicanin I, 11α,13-dihydrohelenalin acetate, chamissonolide, ivalin, and isoalantolactone) **30**, **32**, **50**–**53** was realized. Helenalin showed the lowest IC_50_ concentration against the two parasites, 0.051 μM (*T. brucei rhodesiense*) and 0.695 μM (*T. cruzi*), was related to an increase of oxidative stress inside the parasite [67]. Jimenez et al. proposed that helenalin (**30**) and dehydroleucodine (**31**) acted in *T. cruzi* induced programmed cell death of replicative epimastigotes and infective trypomastigotes [68].

New antitrypanosomal SLs have been isolated through bioassay-guided techniques from the organic extract of *Smallanthus sonchifolius*, which provided three active compounds against epimastigotes of *T. cruzi*, enhydrin (**54**) (IC_50_ 0.84 μM), uvedalin (**55**) (IC_50_ 1.09 μM), and polymatin B (**56**) (IC_50_ 4.90 μM), of which only the first two compounds presented activity against *T. cruzi* trypomastigotes 33.4 μM and 25.0 μM, respectively, being proposed as lead molecules to develop new drugs for Chagas disease [69]. By this same methodology, peruvin (**28**) and psilostachyin (**29**) were tested with *T. cruzi* epimastigotes and gave low values of IC_50_ 6.2 μM and 4.4 μM, respectively, where **29** was more active on the trypomastigotes with an IC_50_ of 2.7 μM compared with 200 μM reached by **28** [59].

The compound 11,13-dehydrocompressanolide (**34**), a guaianolide isolated from *Tanacetum parthenium*, showed activity against multiplicative epimastigotes (IC_50_ 18.1 μM), amastigotes (IC_50_ 66.6 μM), and trypomastigotes (EC_50_ 5.7 μM) of *T. cruzi* (EC_50_: concentration that lyses 50% of the parasites), and a synergistic effect against epimastigotes in the combinational treatment with benznidazole was observed [70]. Recently, two other SLs with similar structures presented an additive effect on the activity against *T. cruzi* acting on different targets. Psilostachyin (**29**), from *Ambrosia tenuifolia*, interacts with hemin, while psilostachyin C (**57**) (*Ambrosia scabra*) inhibits the synthesis of sterols; in the two cases, the parasite death is caused by apoptosis after 4 h of treatment [71].

Otherwise, from a screening of 1800 plant and fungal extracts with in vitro activities against malaria, Chagas disease, and human African trypanosomiasis (two strains) parasites, cynaropicrin (**58**) was considered as a promising molecule for the development of a new antitrypanosomal drug due to the low values of IC_50_ presented for *T. brucei rhodesiense* (IC_50_ 0.3 μM) and *T. brucei gambiense* (IC_50_ of 0.2 μM) [72]. Later, the activity of this SL together with psilostachyin (**29**) against *T. cruzi* was tested in vitro and in vivo. Cynaropicrin had a similar effectivity as the control (benznidazole) against trypomastigotes in vitro; however, the in vivo testing was unable to suppress parasitemia or protect against mortality of the infected mice [73].

In another study, deoxyelephantopin (**59**) showed an IC_50_ value of 0.070 μM against *T. brucei rhodesiense* after being isolated by ethyl acetate partition from *Elephantopus scaber Linn* methanolic extract, which presented the highest activity value from 70 extracts screened from Malaysian plants [74].

### 3.3. Sesquiterpene Lactones with Anti-Schistosoma Activity

In the same way, some sesquiterpene lactones have been studied in the search for alternative drugs to manage schistosomiasis. Vernodalin (**60**), obtained from *Vernonia amygdalina*, showed in vitro activity against *S. japonicum* and inhibited movement and egg laying at 0.055 μM, while another three SLs **61**–**63** from the same species only presented activity at 0.5 μM. Vernodalin was lethal in infected mice with *S. japonicum* at doses up to 5 mg (oral administration), while parasites are not affected at a non-lethal dose of 2.5 mg [75].

Goyazensolide (**64**), a heliangolide extracted from *Eremanthus goyazensis*, shows antischistosomal activity in vitro, and reduced the motility of the worms of *S. mansoni* and caused 90% mortality in the first 24 h of exposure with 9.7–11.0 μM. Similarly, female schistosomas were significantly more susceptible than male worms under the same culture conditions and the compound showed a dramatic reduction of egg production in concentrations up to 2.2 μM [76]. In addition, thapsigargin (**65**) a SL isolated from *Thapsia garganica*, inhibits *S. mansoni* ecto-ATPdiphosphohydrolase (*K*_i_ = 20 μM) [77].

Artemisinin (**66**), an SL present in *Artemisia annua*, in addition to its antimalarial activity, has shown schistosomal activity. Teguments worms were altered in males and females, 30 or 45 days postinfection with *S. mansoni* in mice that were treated for 15 days with 300 or 500 mg/kg of artemisinin [78]; however, due to artemisinin’s low solubility, its derivatives (artemether, dihydroartemisinin, artesunate, and DW-3-15, **67**–**70**) have been studied more often and has shown positive results against *S. haematobium*, *S. japonicum*, and *S. mansoni* [79]; thus, these drugs are show positive results against schistosomiasis and are alternative therapies to praziquantel [80,81].

## 4. CADD Studies against NTDs

**Figure 4:**
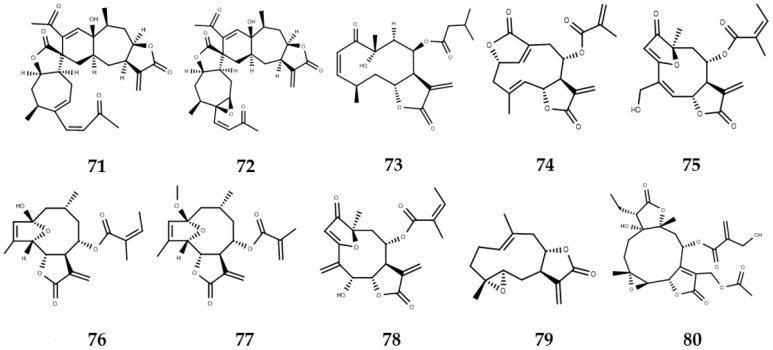

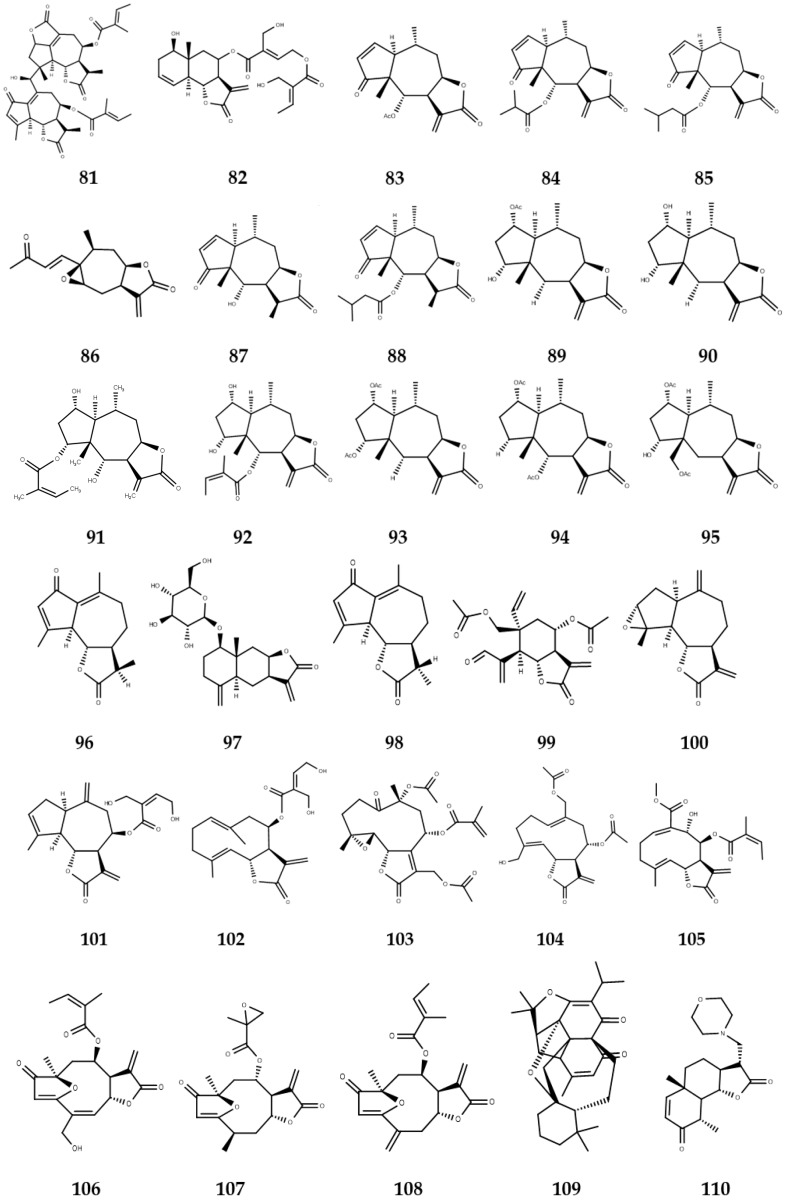
Sesquiterpene lactones studied by in silico methodologies.

In addition, CADD studies have correlated biological activities of some sesquiterpene lactones against NTD-causing parasites (*Trypanosoma* and *Leishmania*) with their chemical structure and developed QSAR models using topological and three-dimensional descriptors. Similarly, there are molecular docking studies testing the interactions of these secondary metabolites (Figure 3 and Figure 4) with target enzymes. Some of these models are purely explanatory and have helped to increase the knowledge of the SLs with respect to their action mechanism, identification of pharmacophore groups, and increase of the biological activity of these molecules. However, it is important to highlight those in silico studies which have found promising molecules with anti-parasitic activity from highly predictive QSAR models and its subsequent experimental validation such as the discovery of 4,15-isoatripicolide tiglate, a SL which possesses the highest in vitro activity against *T. brucei rhodesiense* [82].

### 4.1. Anti-Leishmania In Silico Studies

A series of 40 SLs with activity against *T. brucei*, *T. cruzi*, *L. donovani*, and *P. falciparum* together with their cytotoxicity activities (L6) was evaluated through use of Hologram QSAR (HQSAR) models with 16 series of fragment distinctions and fixed fragment size (four to seven atoms). The PLS models generated were evaluated through cross-validation (leave-one-out and leave-n-out) and external validation, using different latent variables. The five best HQSAR models presented values between 0.637 ≤ Q^2^_LOO_ ≤ 0.78 and 0.653 ≤ *r*^2^_ext_ ≤ 0.944, being the highest coefficients for *L. donovani* [83].

All models using fragment distinction were based on atoms and connections; however, only the activity against *L. donovani* and L6 cytotoxicity is clearly influenced by chirality, H-bond donor, and acceptor group parameters. Afterwards, fragment distinction was fixed and fragment size was varied in eight groups (each 1 of 3 atoms), for the five best models, from 1 to 4 atoms to 8 to 11 atoms [83].

The α,β-unsaturated groups such as 2-methylen-γ-lactone and cyclopentanone are fundamental for the biological activity, as was previously observed [84]. The presence of an epoxide group affects the activity against *T. brucei.* Molecules with a methylcycloheptane ring present higher levels of biological activity, and these values are related to cellular permeation mechanisms. Meanwhile, six-member rings have a relationship with the toxicity by decreasing the protozoal activity at the same time while increasing the toxicity. These results show that it is possible to obtain information respect to the evaluated molecular fragments and the relationships of these with the antiprotozoal and cytotoxic activity observed [83].

A set of 123 sesquiterpenes with leishmanicidal activity, which was subdivided according to their skeleton as sesquiterpene-coumarins, agarofurans, drimanes, pseudoguaianolides, germacranolides, guaianolides, eudesmanolides, and xanthanolides, was studied by molecular docking against four target enzymes: pteridine reductase (*PTR1*, PDB: 2QHX), cysteine synthase (*LmCS*, PDB: 4AIR), trypanothione synthetase (*TryS*, PDB: 2VOB) of *L. major*, and *N*-myristoyl transferase (*NMT*, PDB: 2WUU) of *L. donovani* to obtain more molecular details of the binding modes of these types of metabolites [85].

Some sesquiterpene lactone skeletons presented low docking energy values with the tested enzymes. For PTR1, two xanthanolides, pungiolide A (**71**, Figure 4) and pungiolide B **72**, Figure 4) exhibited the lowest values of docking (−10.6 kcal/mol), with even lower values than the control used (DB07765, −10.2 kcal/mol). Additionally, a similar situation was observed with respect to *LmCS*. the docking energy value of ungiolide A was one of the lowest (−11.4 kcal/mol). In addition, the principal component analysis revealed a higher affinity of neurolenin A (**73**, Figure 4, a germacranolide) for *LmCS*, concluding that these compounds are promising leads for further studies toward the inhibition of the studied enzymes [85].

Recently, in vitro leishmanicidal activity against promastigotes of *L. amazonensis* and *L. braziliensis* of 17 sesquiterpene lactones isolated from five species of the tribe Vernonieae (Asteraceae) was evaluated. An energy minimization of the whole set of molecules was performed by a semi-empirical PM3 method in MOPAC software. With the IC_50_ values, three quadratic QSAR models were obtained where descriptors related to the partition coefficient and polarizability (bpol) presented good correlation with the experimental activities *r^2^* = 0.82 (CLogP), 0.81 (ALogP) and 0.83 (bpol) [86]. The authors, however did not report any type of validation such as cross-validation or external test.

The compounds with the best leishmanicidal activity IC_50_ 1.45 µM) against *L. braziliensis*, isodeoxyelephantopin (**74**, Figure 4), deoxyelephantopin (**58**, Figure 4, IC_50_ 1.34 µM) and *L. amazonensis*, centratherin (**75**, Figure 4, IC_50_ 1.60 µM) showed similar bpol values of 34.9, 32.5, and 34.8 cm^3^, respectively. These results might be related to the molecules’ penetration through the cellular membrane of the parasite and the receptor–ligand recognition where the van der Waals interactions are involved. Likewise, the six most active compounds against the two strains, the three abovementioned compounds together with 2-hydroxy-2,5-epoxy-8-angeloxygermacra-3*Z*,11(13)-dien-6,12-olide (**76**, Figure 4), 2-methoxy-2,5-epoxy-8-methacryloxygermacra-3*Z*,11(13)-dien-6,12-olide (**77**, Figure 4), and isocentratherin (**78**, Figure 4), showed a small range of log *p* values (1 to 3), suggesting that hydrophobicity-specific conditions are necessary for those sesquiterpene lactones to display better antiprotozoal effects [86].

Ogungbe et al. evaluated 28 eudesmanolide-type, 25 guaianolide-type, and 29 germacranolide-type SLs in a molecular docking study of terpenoids with several enzymes of *Leishmania.* The germacranolide-type structures exhibited the lowest docking energies with the *Leishmania* enzymes tested. 4α,5β-Epoxy-8-*epi*-inunolide (**79**, Figure 4) presented the lowest docking energy for *L. major* methionyl t-RNA synthetase (*Lmaj*MetRS) and *L. major* dihydroorotate dehydrogenase (*Lmaj*DHODH) being selective for these two enzymes. 16,17-Dihydrobrachycalyxolide (**80**, Figure 4) with docking values below −96.1 kJ/mol showed the lowest docking energy for *Lmaj*MetRS (−152.9 kJ/mol) and *L. mexicana* phosphor-mannomutase (*Lmex*PMM) (−136.3 kJ/mol), but it was less selective [87].

Interaction of the guaianolides and eudesmanolides was stronger with *Lmaj*MetRS and *Lmaj*DHODH. In overall terms, SLs with the lowest docking energies were observed with *Lmaj*DHODH due to the presence in the active site of a sulfur atom of the cysteine-131 and hydroxyl group of serine-69 that favor the formation of covalent bonds with the electrophilic carbon of the α-methylene lactone groups. Some guaianolides also presented very low values of docking, such as is the case of diguaiaperfolin (**81**, Figure 4) with *Lmaj*DHODH (−154.5 kJ/mol) and *Lmaj*UGPase (−151.8 kJ/mol) and 8β-[4-hydroxy-5-(5-hydroxytigloyloxy)tigloyl]santamarin (**82**, Figure 4) exhibiting selectivity to *Lmaj*MetRS (−153.1 kJ/mol) [87].

### 4.2. Anti-T. brucei and Anti-T. cruzi In Silico Studies

Schmidt et al. tested the in vitro activity of 40 sesquiterpene lactones (16 pseudoguaianolides, four xanthanolides, four modified xanthanolides, eight eudesmanolides, and eight germacranolides) against four protozoan pathogens—*T. brucei*, *T. rhodesiense*, *T. cruzi*, and *L. donovani—*as well as *Plasmodium falciparum* and the cytotoxic activity against L6 rat skeletal myoblast cells. QSAR studies were performed with those data using molecular modeling and multivariate data analysis tools to evaluate the variance in the biological activity in terms of the relative influences of various molecular descriptors. Helenalin (**30**, Figure 3) and its derivatives **83**–**85** (Figure 4) show high activity against the parasites, and even some helenalin congeners have lower IC_50_ values than the used control benznidazole for *T. cruzi*. Another compound, 8-epixanthatin-1,5-epoxide (**86**, Figure 4), a xanthanolide, presents considerable activity against *T. brucei* and *L. donovani* [84].

The influence of presence or absence of α-methylene-γ-lactone and α,β-unsaturated ketone in activity against *T. brucei* and L6-cytotoxicity is studied through the variation in the coefficients of determination (*r*^2^) obtained by multiple linear regression. The whole molecule set generated low *r*^2^ values for *T. brucei* and L6-cytotoxicity (0.61 and 0.41, respectively), and the significance of these two descriptors was confirmed by *F*- and *t*-tests. While only considering 16 related compounds of the helenanolides **30**, **32**, **50**, **51**, **83**–**85**, and **87**–**95** series, higher *r*^2^ values against *T. brucei* and L6-cytotoxicity (0.87 and 0.79, respectively) were achieved. The authors interpreted that in the case of compounds possessing very similar molecular structure, the modulating influence of other structural factors on bioactivity is small compared with the major impact of chemical reactivity (i.e., presence of enone and methylene lactone groups) [84,88,89].

Three QSAR models was evaluated. The AM1 semi-empirical method was initially used to optimize the molecule structure for posteriorly calculating 44 descriptors in the MOE software to generate an initial model. PLS analysis was performed using two dependent variables (L6 cytotoxicity and *T. brucei* activity). The final model contained three latent variables from 20 selected descriptors and presented a Q^2^ value for *T. brucei* and L6 cells of 0.71 (for both), which is significantly higher than the model obtained using the whole set of molecules and seven latent variables from 44 descriptors that presented a Q^2^ value of 0.52 and 0.41, respectively. However, PLS models only were evaluated through internal validation (cross validation, leave-one-out) without any external test [84].

Helenalin (**30**) was also studied computationally through molecular docking using two sirtuins (silent-information regulator 2 proteins), *T. cruzi* Sir2RP1, and Sir2RP3, which act as antiparasitic targets. The PLP score was used to rank and evaluate the docking poses and these were compared to select inhibitors of sirtuin classes I and III, AGK2, and thiobarbiturate 6. PLP docking scores obtained were 40.6 (Sir2RP1) and 49.2 (Sir2RP3), and intermediate values with respect to the best scores were reached by anacardic acid (8E, 11E) of 80.7 for Sir2RP1 and aculeatin D of 90.3 for Sir2RP3 [90].

A purely explanatory QSAR study was performed for 15 sesquiterpene lactones **28**, **29**, **33**, **55**, **59**, **96**–**105** with in vitro activity against *T. cruzi* epimastigotes previously determined. Initially, the geometry of all molecules was pre-optimized through Molecular Mechanics Force Field (MM+) and refined by the semi-empirical AM1 method. A set of one-dimensional (1D), two-dimensional (2D), and three-dimensional (3D) descriptors was calculated through PaDEL 2.11. Additionally, Weighted Holistic Invariant Molecular (WHIM) 3D molecular descriptors used (*x*, *y*, *z*) coordinates of a molecule were used within different weighting schemes to generate a value that represents its structural features with respect to size, shape, symmetry, and atom distribution [91]. The QSAR model was evaluated by internal validation, (cross validation, leave-one-out) and determination coefficient *r^2^*. SLs studied were divided in two groups, a training group (composed by 12 SLs) and a test group (conformed by **28, 29,** and **59**) whose selection method was not reported. pIC_50_ values predicted by the best QSAR model (*r^2^* = 0.99), which relates two descriptors, LAC (present or absence of α,β-unsaturated lactones) and W_vol_ (a holistic descriptor related to steric factors), are close to the experimental values reported for these 3 SLs. Cross-validation coefficient values are not reported. The authors found that a correlation of the observed antitrypanosomal activity existed with the steric parameters of the set of molecules tested and concluded that SLs have a high potential as lead molecules against Chagas disease [92].

An excellent example of the discovery of new compounds with high antiparasitic activity from in silico researches was found from the prediction of in vitro activity of 1750 SLs structures of a virtual library through a QSAR model performed with 69 molecules. Because of the high levels of activity against *T. brucei rhodiense*, furanoheliangolide-type compounds appear as interesting leads against human African trypanosomiasis. From the three-dimensional structures of the 69 molecules, whose energies were minimized through AM1 semi-empirical method, 123 molecular descriptors were calculated. The whole set of molecules was divided into a training set (46 molecules) and test set (23 molecules), and this division was based on a structural diversity classification. A QSAR model generated, which is correlated with the presence of reactive structure elements and their electronic properties, is important to the hydrophobic volume balance, and is codified by VolSurf descriptors with a significant contribution in the model [93]. Internal validation (cross-validation, leave-one-out) presented a 0.65 Q^2^ value [82].

In the prediction of activity against *T. brucei rhodesiense*, 71 molecules presented pIC_50_ values greater than or equal to 7.0, and 15 compounds corresponded to furanoheliangolide-type SLs. Therefore, the antitrypanosomial activities of four of these were evaluated, goyazensolide (**64**, Figure 3, predicted pIC_50_, 7.62), budlein A (**106**, Figure 4, predicted pIC_50_, 6.60), and 4,5-dihydro-2′,3′-epoxy-15-deoxygoyazensolide (**107**, Figure 4), which presented a lower activity than the others according to the authors due to hydrogenation of the conjugated γ,δ-double bond between C-4 and C-5 (predicted pIC_50_, 6.17) and 4,15-isoatripicolide tiglate (**108**, Figure 4, predicted pIC_50_, 7.04)**,** and experimental pIC_50_ values of 7.14, 7.14, 6.70, and 7.82, respectively, were obtained. The QSAR model underestimated the activities of budlein A and 4,15-isoatripicolide tiglate, while it overestimated goyazensolide [82].

From this QSAR model and subsequent validation through in vitro tests performed, furanoheliangolide (**108**, Figure 4) 4,15-isoatripicolide tiglate was found as the most potent sesquiterpene lactone against *T. brucei rhodesiense,* with a IC_50_ value of 15 nM and a selectivity index of 77 (cytotoxic assay was performed with L6 rat skeletal myoblasts), which is an interesting lead against *T. brucei rhodesiense* [82].

Finally, an interesting study was performed by Hu et al. who tested the potential adverse effects of 89 natural products and derivatives (including some SLs) with activity against *Trypanosoma* in VirtualToxLab, an in silico tool for estimating the toxic potential of drugs, chemicals, and natural products. The results obtained showed a wide range of potential toxicity, from 0.161 classified as benign of germacranolide (**109**, Figure 4) to 0.593, potentially harmful, of compound **110**, indicating a moderate risk to develop adverse effects, which might be due to continued exposure to some of the 89 compounds evaluated. In addition, a fraction of the tested compounds bound to nuclear receptors, favoring the development of endocrine dysregulation, while other compounds showed enzyme inhibition of the cytochrome P450 family [94].

## 5. Conclusions and Perspectives

In this review, we have covered the problematic aspects of the chemotherapies currently used against leishmaniasis, Chagas disease (American trypanosomiasis), sleeping sickness (human African trypanosomiasis), and schistosomiasis, and we showed how natural products have emerged as alternatives for the development of new medications against these NTDs. Specifically, sesquiterpene lactones, and secondary metabolites characteristic of the Asteraceae family have shown promising in vitro and in vivo activity, and some promising molecules have emerged from these studies.

Recently, CADD studies (mainly molecular docking and QSAR) have appeared with the aim to obtain new molecules by modify some existing molecules and understanding the mechanism of action of the SLs, which also increases the knowledge of this topic. In vitro tests for confirming the mechanism of action of the most active selected SLs in the docking studies here referenced were not reported. Hence, the obtained information from those studies are inconclusive regarding its enzymatic activities and demand further study.

This research area is still developing and an example of that is the case of schistosomiasis, where there have been no in silico studies with SLs. Therefore, it is important to continue performing CADD studies to find solutions with low cost and few side effects for the health and social problems caused by NTDs.
